# Date Pit Carbon Dots Induce Acidic Inhibition of Peroxidase and Disrupt DNA Repair in Antibacteria Resistance

**DOI:** 10.1002/gch2.201900042

**Published:** 2019-08-27

**Authors:** Ayan A. Nurkesh, Qinglei Sun, Haiyan Fan, Kanat Dukenbayev, Andrey Tsoy, Akerke Altaikyzy, Kunjie Wang, Yingqiu Xie

**Affiliations:** ^1^ School of Sciences and Humanities Nazarbayev University Nur‐Sultan 010000 Kazakhstan; ^2^ Key Laboratory for Applied Technology of Sophisticated Analytical Instrument of Shandong Province Shandong Analysis and Test center Qilu University of Technology (Shandong Academy of Sciences) Jinan 250014 China; ^3^ School of Engineering Nazarbayev University Nur‐Sultan 010000 Kazakhstan; ^4^ National Laboratory Astana Nazarbayev University Nur‐Sultan 010000 Kazakhstan; ^5^ College of Petrochemical Technology Lanzhou University of Technology Lanzhou 730050 China

**Keywords:** bacteria drug resistance, carbon nanodots, nanobiology, nanozymes, pH, Pim‐1

## Abstract

Carbon nanodots (C‐dots) are emerging as a new type of promising agent in anticancer, imaging, and new energy. Reports as well as the previous research indicate that certain C‐dots can enhance targeted cancer therapy. However, in‐depth mechanisms for such anticancer effect remain unclear. In this work, treatment provided by the date pit‐derived C‐dots, exhibits significant DNA damage; Annexin V/7‐AAD‐mediated apoptosis, and G2/M cell cycle arrest in prostate cancer cells. The application of C‐dots to the cell generally leads to acidulation of the cell medium, cooperated with membrane compact. The date pit‐derived C‐dots are observed inhibiting the horseradish peroxidase. Moreover, the C‐dots disrupt likely through nucleotide excision DNA repair at low dose during DNA ligation step suggesting the antimicrobial effect and targeting Pim‐1, EGFR, mTOR, and DNA damage pathways in cancer cells. For the first time the detailed and novel mechanisms underlying the C‐dots, derived from the date‐pit, as an efficient, low‐cost, and green nanomaterial are reveled for cancer therapy and anti‐infection.

## 1 Introduction

Recently carbon‐based nanomaterials including C‐dots have attracted much attention in the fields of biomedicine.^1^, ^2^ C‐dots generally show the high stability, biocompatibility, and simplicity of synthesis. The C‐dots can be derived from a variety of resources and different types of doping are used to enhance specific properties of C‐dots.^3^, ^4^, ^5^, ^6^, ^7^, ^8^ Recent studies showed that food‐derived C‐dots exhibit inhibition against cancer cell growth. For instance, C‐dots synthesized from ginger inhibit HepG2 cells growth in vivo of mouse model.^9^ C‐dots derived from green tea inhibit the growth of MCF‐7, MDAMB‐231 breast cancers, and prostate cancer cells as we and others showed.^10^, ^11^ C‐dots derived from date pits (*Phoenix dactylifera*) inhibit cancerous cell migration with changes in cytoskeleton.^12^ Mechanistically, tea derived C‐dots were found to inhibit cancer cell growth and interact with nuclear protein ARF and regulate hippo pathway.^11^ As regular therapies may easily induce drug resistance, there is a huge demand for an alternative approach. Therefore, people attempted to use nanoparticles in drug delivery.^13^ Given that C‐dots with their small average size, and small enough to be able to enter into nucleus,^11^ they may potentially interact with DNA leading to the DNA damage induced cell death. On the other hand, they may cause the mediated signaling cascade. Moreover, nanoparticles may potentially act like enzyme in many biological chemical reactions, named as nanozyme,^14^, ^15^, ^16^ though whether C‐dots also have the nanozyme‐inhibitor activity is unclear. Herein, we tested the function of C‐dots in peroxidase activity with pH‐mediated regulation and their potential to react with reactive oxygen species (ROS). Thereafter, we will study the role of date pit‐derived C‐dots in the apoptosis and cell cycle arrest in cancer cells to reveal their action in enzymatic and acidic regulation. Moreover, C‐dots have been widely recognized with their antibacterial effects.^17^, ^18^, ^19^, ^20^, ^21^, ^22^, ^23^ In this work, we attempted to identify the antibacterial effect potentially brought by date pit‐derived C‐dots, and explore the mechanism. Finally, we will estimate the potential of date pit‐derived C‐dots‐mediated DNA damage and repair pathways, cell proliferation signaling using mutation or cancer cell lines.

## 2 Results and Discussion

**Figure 1:**
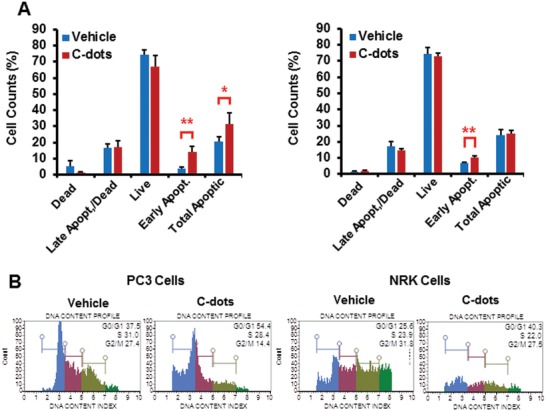
Date pit‐derived carbon nanodots induce distinct apoptosis, and cell cycle arrest and DNA damage in cells. A) Prostate cancer PC3 and NRK cells were treated by C‐dots (0.2 mg mL^−1^) for 16 h and cells were collected and subjected to flow cytometry using Annexin V/7‐AAD staining kits (Millipore) or cell cycle analysis B) using staining kits (Millipore).

The as prepared C‐dots were applied to the prostate cancer PC3 cells and normal rat kidney (NRK) cells in cytotoxicity assay. As shown in **Figure** **1**, C‐dots developed significant total apoptosis induced by Annexin V/7‐aminoactinomycin D (7‐AAD) in PC3 cells compared to vehicle treated control, whereas NRK cells showed very little sensitivity to C‐dots in terms of total apoptotic cells. Interestingly, both PC3 and NRK cells showed the early apoptosis events upon C‐dots treatment suggesting that there is a common target of C‐dots in both normal and cancerous cells. However, no significant difference was observed in the population change upon C‐dots treatment in late‐stage apoptotic cells between PC3 and NRK measured by the impermeant dye, 7‐AAD, indicating this mechanism is not dominated in the total apoptosis. Cell cycle analysis showed that C‐dots significantly induced both G0/G1 and G2/M arrest in PC3 cells whereas only G0/G1 arrest in NRK cells (Figure 1B), suggesting PC3 cells are more sensitive to cell cycle inhibition by C‐dots. Thus, date pit‐derived C‐dots may significantly inhibit prostate cancer cell growth and induce cell cycle arrest with total apoptosis while leaving the normal cells nearly intact. Our data suggest that such C‐dots may be developed into potential anticancer agents.

**Figure 2:**
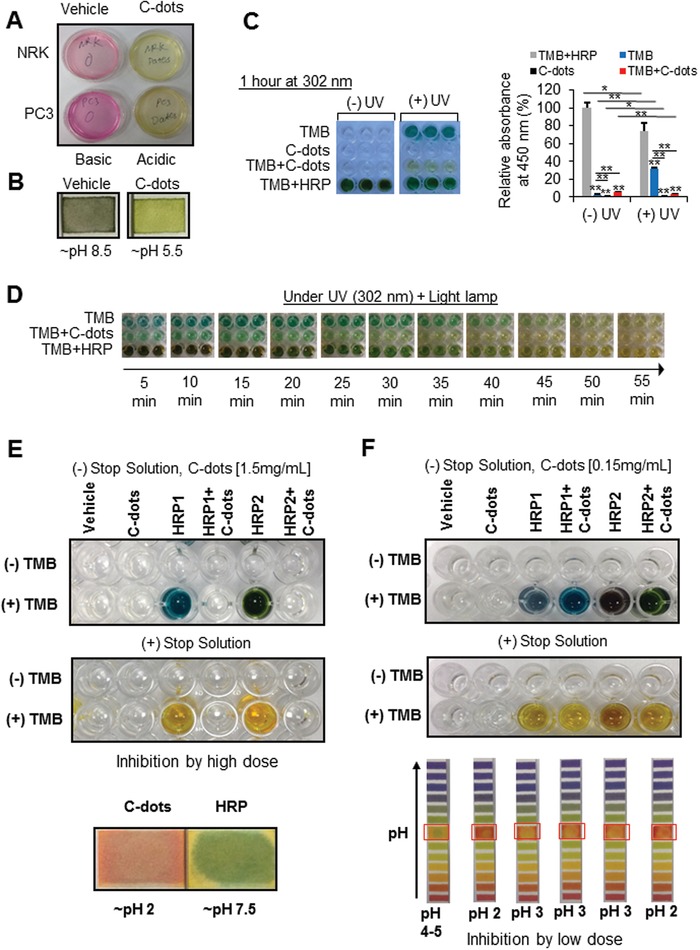
Date pit‐derived carbon nanodots inhibit peroxidase, ROS by affecting pH. A) pH of cultured cell medium decreases upon C‐dots treatment (0.05 mg mL^−1^). B) Date C‐dots inhibit ROS generation in both NRK and PC3 cells. C) Date C‐dots exhibit UV‐induced peroxidase and inhibition activity. D,E) Date C‐dots inhibit peroxidase activity with decreased pH at low and high concentrations using HRP as peroxidase enzyme control and TMB as substrate.

The treatment of C‐dots causes a significant color change of the medium from pink to yellow shown in **Figure** **2**A for both PC3 and NRK cells. Indicated in Figure 2B, the color change is the result of pH change from pH 8.5 to 5.5 upon treatment with C‐dots. We observed that 3,3′,5,5′‐tetramethylbenzidine (TMB) was able to be oxidized under the UV irradiation. Depicted in Figure 2C, UV irradiation leads the TMB to the blue color just like the effect of peroxidase horseradish (HRP) only with lighter tint. The addition of date pit C‐dots to the TMB under UV irradiation showed a yellow color just as the result of adding the stop buffer. When we put TMB, TMB‐C‐dots, and TMB–HRP all under UV irradiation, the effects were observed versus time, and the results are summarized in Figure 2D, wherein TMB‐C‐dots system reaches the equilibrium within 30 min, TMB–HRP system 45 min, and TMB only 50 min. Consequently, we observed in Figure 2E that the oxidation of TMB by HRP was suppressed by the presence of C‐dots. We observed different tone of blue for the mixture of TMB and HRP with the addition of C‐dots. After adding the stop solution, we then observed the yellow color in different tones. Indicated in Figure 2F, the fully suppression by C‐dots is observed at high concentration of C‐dots. Such kind of peroxidase suppression is analogous to what happens for the peroxidase occurring in the strong acidic media. Therein, the fundamental cause for the peroxidase suppression observed here is the acidity brought by the C‐dots.^24^ It is then hypothesized the as prepared C‐dots may inhibit peroxidase through acidulation. Our data suggest that the C‐dots have nanozyme activity of peroxidase and can also compete binding to substrate with HRP.

**Figure 3:**
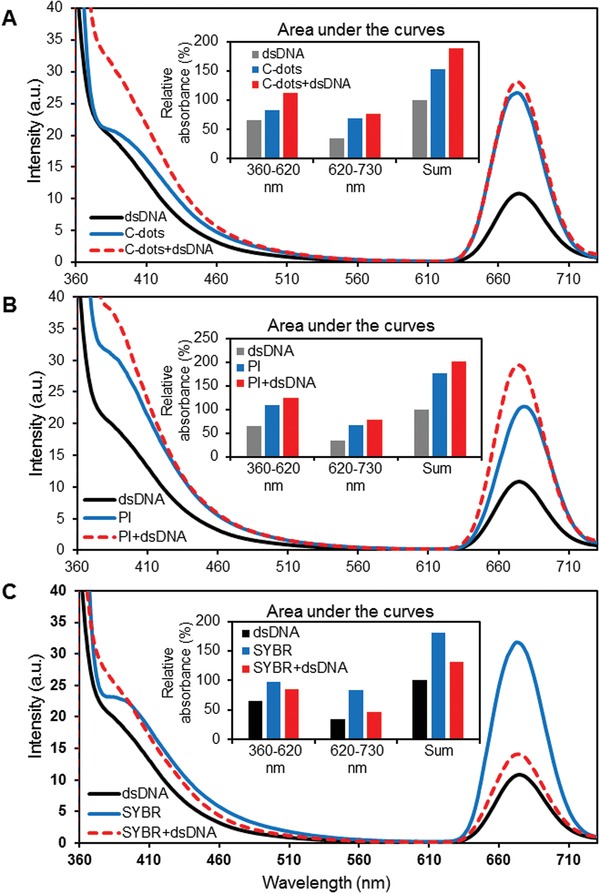
Date pit‐derived carbon nanodots compete with DNA binding dyes on DNA interactions. SYBR safe and PI were applied for the competitive assay. Double‐stranded DNA of plasmid was used for analysis.

Given that the C‐dots induced DNA damage in cells, to further identify the nature of the binding between DNA and C‐dots, we performed a series of fluorescence measurement of double strand DNA (ds‐DNA), C‐dots, and the combination of ds‐DNA and C‐dots. Excited at 340 nm, two fluorescence bands were observed at 400 and 670 nm for both DNA and C‐dots. Depicted in **Figure** **3**A, the combination of ds‐DNA and C‐dots leads to a nonadditive fluorescence enhancement. We then used two other systems ds‐DNA and propidium iodide (PI) (Figure 3B) as well as ds‐DNA and SYBR (Figure 3C). The fluorescence of the combination of PI and ds‐DNA is, not just the simple combination of the fluorescence of DNA and C‐dots, nevertheless stronger than the fluorescence of PI and ds‐DNA only. Shown in Figure 3C, the fluorescence of SYBR was quenched with the addition of double strand DNA. Interestingly, both PI and SYBR absorb at 340 nm and their fluorescence behavior is quite similar to that of diluted DNA and date pit C‐dots. The overall fluorescence properties of the combination of ds‐DNA and C‐dots is analogous to that of ds‐DNA and PI indicating C‐dots potentially bind to ds‐DNA through intercalate binding, in other word, C‐dots might bind to ds‐DNA through the hydrogen bonding with the bases. It is well known that PI binds ds‐DNA through intercalate binding, whereas SYBR binds ds‐DNA likely through minor groove. The comparison among three systems indicate that C‐dots potentially bind to ds‐DNA through intercalate binding, in other word; C‐dots might bind to ds‐DNA through the hydrogen bonding with the bases.

Cytoplasmic pH has been found critical in the determination of cell growth, proliferation, and cell death.^25^ Apoptosis in various cancer cells is found sensitive to the cytoplasmatic pH.^26^, ^27^, ^28^, ^29^ However, it remains unknown whether the change in the intercellular pH is the cause or the consequence of the apoptosis. The observation in the acidity change in the present study is actually consistent with the fluorescence spectra where ample evidences reveal the innegligible interaction between C‐dots and double strand DNA. Most likely this interaction leads to the DNA damage and consequently, the inhibition of cancer cell growth through selective cell cycle arrest and therefore enhanced apoptosis. The different sensitivity toward the date pit C‐dots by the normal and cancer cell in terms of cell apoptosis can be attributed to the fundamental difference in the membrance structure between the normal and the cancer cell. It has been long recoganized that the surface of cancer cell is unevenly distributed with the particles of the size from 30 to 300 Å, whereas the surface of the normal cell is rather evenly distributed with the particles of the size from 30 to 60 Å.^30^ Such membrane structure difference determines that the cancer cells prefer to stay isolated and migrating. Moreover, the uneven distribution of particles with various size leads to some weak stress on the surface of the cancer cell that are prone to the attack by C‐dots, especially when C‐dots show strong affiliation to the cell membrane.

**Figure 4:**
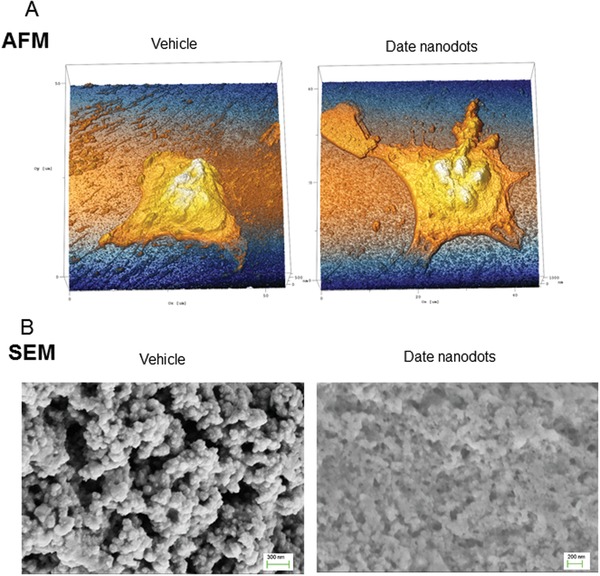
Date pit‐derived carbon nanodots induce changes in cell surface tested by single molecular A) high resolution AFM and B) SEM. PC3 cells were fixed by ice cold methanol after treatment with the C‐dots or PBS as vehicle control and cell morphology was investigated. AFM, atomic force microscopy; SEM, scanning electron microscopy.

In order to examine the effect of date pit‐derived C‐dots on cell apoptosis, we collected the images of the affected cells using high‐resolution surface atomic force microscopy (HR‐AFM) and scanning electron microscopy (SEM). Using PBS vehicle as control, C‐dots treated cells showed dramatic morphology changes on cell surface, in particular, more filopodia and lamellipodia structures developed on the membrane (**Figure** **4**A). The filopodia and lamellipodia formation are likely induced by nanoparticles physically interacting with cell membrane, indicated as some hollow structures on surface, which match the size of nanoscale of 10–500 nm on the edge of cells. On the other hand, SEM images (Figure 4B) exhibit a dramatic morphology change upon application of the C‐dots. The C‐dots lead to a structural collapsing accompanied with a boundary disappearing. Both AFM and SEM images indicate the cell death was induced by the rapture of cell membrane, and the destruction of the cell membrane is most likely caused by the strong interaction between C‐dots and cell membrane. In addition, the cells were found resistant to trypsin digestion during the cell culture. Such resistance is believed to be due to the cell morphology change originated by the formation of filopodia and lamellipodia which stop cells from dividing and growing. In summary, date pit‐derived C‐dots have shown great potential in the inhibition of cancer cells, which makes such C‐dots promising for clinical application in cancer therapy through nanozyme activity and pH changes.

**Figure 5:**
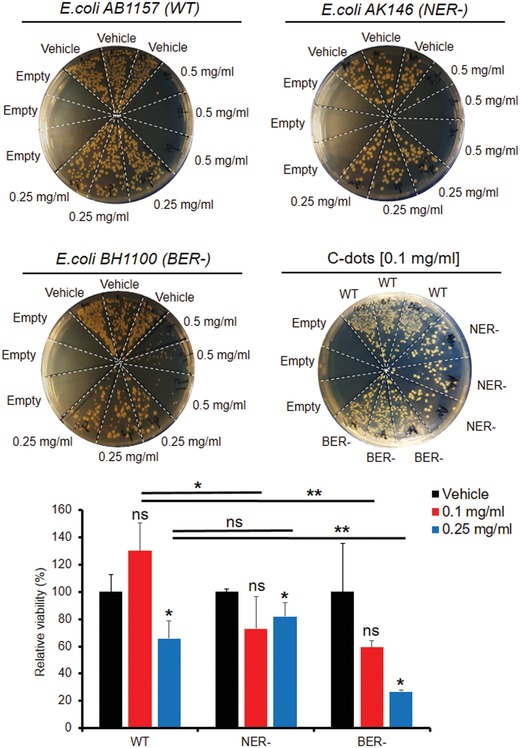
Date pit‐derived carbon nanodots disrupt base excision repair using bacteria mutation (−) model system. BER: base excision repair; NER: nucleotide excision repair; WT: wild type. Bacteria were cultured overnight followed by dilution and aliquot to be subjected to treatment with concentrations of the C‐dots indicated. Finally, cells were diluted and replated and colonies or OD were counted after overnight growth on LB agar plates.

To further explore the potential effect and mechanisms of the C‐dots on DNA, we applied a bacteria DNA repair system. The antimicrobial effect of the date pit‐derived C‐dots against three bacteria strains *Escherichia coli* AB1157 (WT), AK146 (NER mutation), and BH1100 (BER mutation) with/without UV radiation was tested and the results were presented in **Figure** **5** indicating the C‐dots concentration dependence of the antibacterial effect and DNA repair. At the C‐dots concentration of 0.5 mg mL^−1^, WT and NER exhibit much lower viability than BER. For NER, the C‐dots concentration of 0.1 mg mL^−1^ achieves lower cell viability than 0.25 mg mL^−1^. While all the evidences indicate that the bacterial cell death is the consequence of cell membrane rapture. It is then reasonable to assume the interaction between C‐dots and cell membrane plays an important role in the antimicrobial effect, wherein the lower viability is usually achieved by providing most amount of activity sites.^31^ While at the concentration of 0.5 mg mL^−1^, although the aggregation among the C‐dots is common, the overall number of the activity centers is large, however, at the concentration of 0.25 mg mL^−1^, the aggregation among C‐dots may lead to a reduction of the overall activity centers that C‐dots may provide. This leaves the concentration of 0.1 mg mL^−1^ with more activity centers than 0.25 mg mL^−1^. Nevertheless, the interaction with cell membrane does not take sole responsibility for the bacterial cell fetal. As the results, only for NER bacterial strain, 0.1 mg mL^−1^ performs better than 0.25 mg mL^−1^. In the case of WT and BER bacterial strains, factors such as oxidation stress and DNA damage other than direct interaction with cell membrane may also be responsible for the bacterial cell fetal.^32^

As for the role of the UV irradiation, it is generally believed that UV radiation may induce ROS with or without the aid of C‐dots. The observation by our other data generally indicates that NER is most vulnerable toward UV irradiation than BER. Combining our results described earlier that as prepared C‐dots exhibited inhibition against the peroxidase through acidulation, it is now clear that C‐dots counteract the effect of UV irradiation in NER bacterial strain and consequently deter the overall antimicrobial effect. In the case of BER, the bacteria cell death may be the result of both interaction between C‐dots and cell membrane caused by C‐dots and the oxidative stress brought by the ROS induced by UV irradiation.^23^

**Figure 6:**
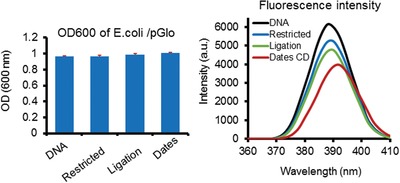
Date pit‐derived carbon nanodots inhibit DNA ligation using plasmid DNA with GFP reporter. GFP reporter plasmid pGLO was digested by restriction enzyme then ligated by T4 DNA ligase followed by transformation of competent cells and measurement of GFP in LB medium culture using OD as control of cell growth.

In the further test, we attempted to study how the C‐dots may affect the DNA ligation during the DNA repair steps. We used DNA digestion of plasmid DNA containing a GFP reporter and T4 DNA ligase‐mediated self‐ligation to test the effect. The C‐dots were found inhibited DNA ligation, which results in the disruption of reporter GFP expression in bacteria (**Figure** **6**).

**Figure 7:**
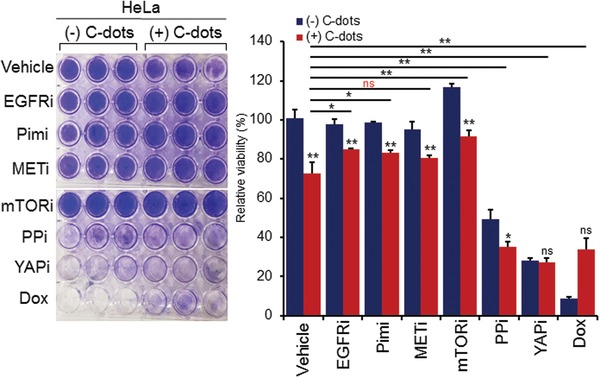
Date pit‐derived carbon nanodots inhibit cancer cell proliferation through mTOR, Pim‐1, DNA damage, and EGFR pathways. The C‐dots at 0.15 mg mL^−1^ were used for combination or singe treatment with/without inhibitors of mTOR, MET, and YAP at 10 × 10^−6^ m or Pim‐1, EGFR at 1 × 10^−6^ m, phosphatase (PPi cocktail 100×, APExBIO, Houston, USA) at 10^5^ dilution and doxorubicin (DOX) at 100 × 10^−6^ m.

To further explore the signaling pathways of the antiproliferation of cancer cells by the C‐dots, we used a inhibitor screening by targeting multiple pathways including kinases of Pim, mTOR, EGFR, MET, and chemotherapeutic drugs of phosphotase inhibitor, Doxorubicin (DOX) in cancer cells of HELA. As shown in **Figure** **7**, the data suggest that kinases of Pim (Pim‐1), mTOR, EGFR (but not MET), and DOX pathways are main signaling the HELA cells sensitize to the C‐dots. When the inhibition of mentioned pathways happens, the combination with the C‐dots results in the cell proliferation against the C‐dots alone effect. The data suggest that the C‐dots may mediate the cell growth inhibition by these pathways including Pim‐1, mTOR, EGFR, and DNA damage.

In summary, we systematically analyzed the C‐dots‐mediated nanozyme, DNA damage, DNA repair, and cell death in both cancer cells and bacteria with novel discovery that the C‐dots induce pH‐mediated nanozyme inhibition, photo‐induced nanozyme activity, destroy of BER‐mediated DNA repair, and kinase‐mediated cell proliferation, which are consistent with our previous hypothesis of MET kinase synergistic DNA damage targeted pathway.^33^

## 3 Conclusion

The date pit‐derived C‐dots cause the apoptosis of PC3 cells while leaving rather modest cytotoxicity against the NRK cells. The application of the date pit‐derived C‐dots was found partially or completely inhibit the peroxidase by HRP due to the acidulation effect brought to cell medium by C‐dots. The apoptosis of PC3 cell was mainly attributed to the rapture of the cell membrane caused by the interaction with C‐dots. Because of the similar interaction between C‐dots and the cell membrane, the as prepared C‐dots exhibit strong antibacterial effect against three bacteria WT, NER, and BER. While the bacterial cell death was attributed to the damage of the DNA and cell membrane as the result of C‐dots attack for WT and NER, the antibacterial effect against BER was believed to be related to the oxidative stress brought by DNA damage with or without the aid of C‐dots. Moreover, the C‐dots disrupt DNA repair through BER pathway and inhibits cancer cell proliferation likely through mTOR, Pim‐1, EGFR, and DNA damage pathways.

## 4 Experimental Section

The synthesis and characterization of the date pit‐derived C‐dots were published in the previous work.^12^ The fluorescence excitation and emission spectra were measured by a Cary eclipse fluorescence spectrophotometer (Agilent Technologies).

For the cell proliferation assay, cells were plated on 24‐well plates in DMEM medium (Life Technologies) with 10% FBS (Life Technologies). Cells were treated for 96 h by various concentrations of C‐dots, followed by staining with Crystal Violet dye for 30 minutes and washing by water, and imaging with OD (optical density) measurement.

In order to measure the percentage of cells in different cell cycle phases, cells were cultured in DMEM medium with 10% FBS for overnight at 37 °C followed by treatment with C‐dots or with PBS vehicle as control. Further steps were transferring cells to eppendorf tubes, centrifugation for 1 min, discarding the supernatant, washing by PBS, and resuspending the pellet in PBS. Next, ice‐cold 70% ethanol (200 proof absolute ethanol which was precooled at −20 °C) were applied to the cells drop by drop, by gentle shaking tubes with fingers, followed by incubation for at least 3 h at −20 °C. Then the cells were washed with PBS once. Finally, cells were incubated with 200 µL of the coloring reagent (Muse Cell Cycle kit), incubated for 30 min at room temperature in the dark, and analyzed with Muse cell analyzer.

For determining the cells early apoptosis, the grown cells were diluted in 1% FBS at a concentration of 1 × 10^6^ to 2 × 10^6^ cells mL^−1^. Next, 100 µL of cells with 100 µL of Annexin V reagent (Muse Annexin V & Dead Cell Reagent) were mixed and incubated for 20 min at 37 °C and measured in a flow cytometer. For cell death assay, PC3 and NRK cells were resuspended in 1% FBS containing medium with a number of 1 × 10^6^ cells followed by the protocol of Muse Annexin V & Dead Cell assay kits measured by the Muse cell analyzer (Merck Millipore).

The peroxidase assay was measured by incubating 100 µL of TMB substrate with dates carbon dots for 30 min. Commercial HRP enzyme was used as control. After the addition of Stop Solution, the light absorbance was measured with microplate reader (Thermo Scientific Varioskan Flash). UV light source was the 302 and 365 nm by UV Benchtop Transilluminator.

The dsDNA binding by the C‐dots was tested by the fluorescent emission of plasmid DNA mixed with SYBR Safe, or PI (Thermo Scientific) measured by Cary eclipse fluorescence spectrophotometer.

For bacteria mutation analysis, overnight culture of cells was diluted and continued incubate with the C‐dots for 3 h then overnight culture on plates for counting of colony. The bacterial strains were described as previous reports.^34^, ^35^, ^36^, ^37^, ^38^ For self‐ligation assay, pGLO plasmid (Biorad) was digested by restriction enzyme then ligated by T4 DNA ligase (NEB) followed by transformation and counting of colony or measurement of GFP in LB medium culture.

## Conflict of Interest

The authors declare no conflict of interest.
